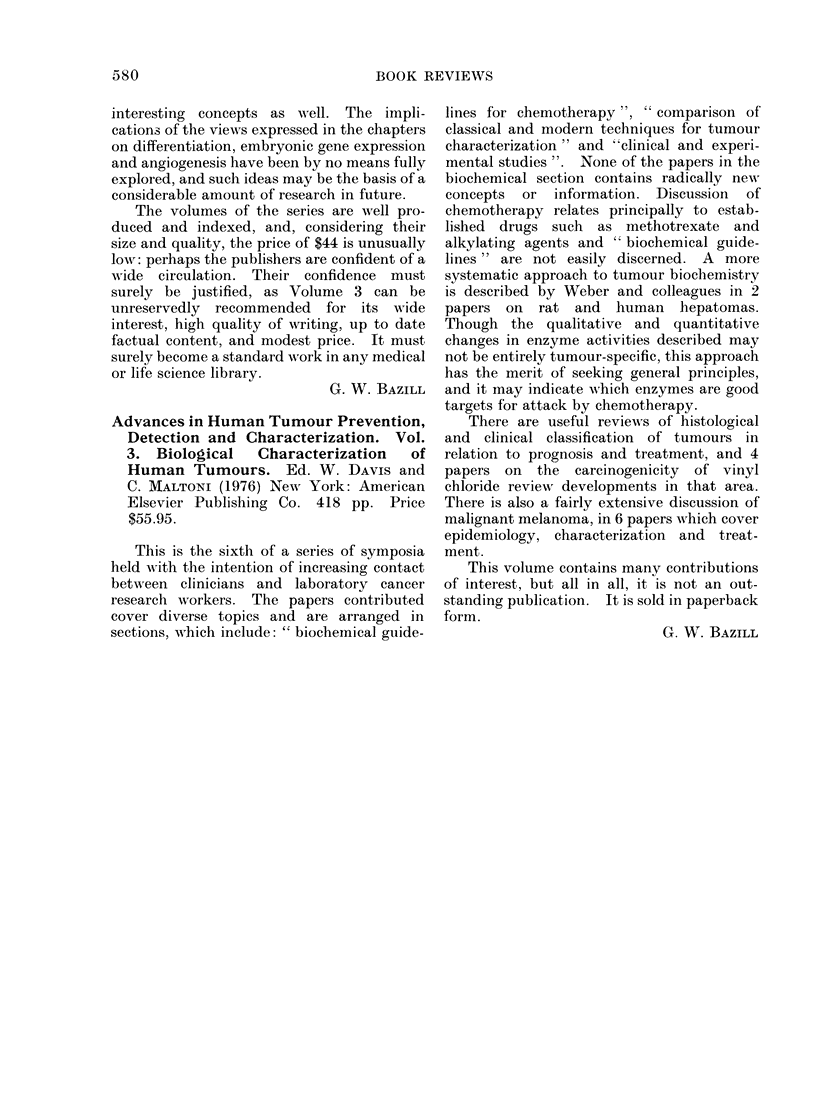# Advances in Human Tumour Prevention, Detection and Characterization. Vol. 3. Biological Characterization of Human Tumours

**Published:** 1976-11

**Authors:** G. W. Bazill


G. W. Bazill

**Advances in Human Tumour Prevention, Detection and Characterization. Vol. 3. Biological Characterization of Human Tumours.** Ed. W. Davis and C. Maltoni (1976) New York: American Elsevier Publishing Co. 418 pp. Price $55.95.

This is the sixth of a series of symposia held with the intention of increasing contact between clinicians and laboratory cancer research workers. The papers contributed cover diverse topics and are arranged in sections, which include: " biochemical guidelines for chemotherapy ", " comparison of classical and modern techniques for tumour characterization " and "clinical and experimental studies ". None of the papers in the biochemical section contains radically new concepts or information. Discussion of chemotherapy relates principally to established drugs such as methotrexate and alkylating agents and " biochemical guidelines " are not easily discerned. A more systematic approach to tumour biochemistry is described by Weber and colleagues in 2 papers on rat and human hepatomas. Though the qualitative and quantitative changes in enzyme activities described may not be entirely tumour-specific, this approach has the merit of seeking general principles, and it may indicate which enzymes are good targets for attack by chemotherapy.

There are useful reviews of histological and clinical classification of tumours in relation to prognosis and treatment, and 4 papers on the carcinogenicity of vinyl chloride review developments in that area. There is also a fairly extensive discussion of malignant melanoma, in 6 papers which cover epidemiology, characterization and treatment.

This volume contains many contributions of interest, but all in all, it is not an outstanding publication. It is sold in paperback form.